# Primary Pericardial Epithelioid Angiosarcoma: A Diagnostic Dilemma: A Case Report

**DOI:** 10.30699/IJP.2021.136440.2494

**Published:** 2020-07-06

**Authors:** Tahmineh Mollasharifi, Behrang Kazeminezhad

**Affiliations:** 1 *Department of Pathology, Shahid Modarres Hospital, Shahid Beheshti University of Medical Sciences, Tehran, Iran*

**Keywords:** Heart tumor, Immunohistochemistry, Pericardium, Vascular neoplasm

## Abstract

The incidence of pericardial epithelioid angiosarcoma is rare. Angiosarcoma of pericardium may coat the pericardium in a diffuse fashion. Diagnosis of an angiosarcoma is challenging and may be easily mistaken as constrictive pericarditis. Herein, a case of primary pericardial angiosarcoma is reported in a 16-year-old female. Patient presented with chest pain and dyspnea on exertion, regarded as constrictive pericarditis. Pericardectomy was performed and histopathologic examination showed pleomorphic epithelioid cells exhibiting hyperchromatic nuclei, prominent nucleoli and eosinophilic cytoplasm arranged in sheets and occasionally lined irregular vascular spaces. Moreover, immunohistochemical staining revealed that tumor cells were positive for CD31 and vimentin. The patient received chemotherapy with adriamycin, ifosfamide, and mesna. Unfortunately, the patient died of cardiac involvement and pleural metastases less than three months following the operation. Primary pericardial angiosarcoma is rare and difficult to diagnose, especially epithelioid variant. Immunohistochemical assessment is required to confirm the final diagnosis.

## Introduction

Angiosarcoma, despite being the most common primary malignant tumor of the heart and pericardium, is itself an extremely rare tumor with a 0.001 to 0.003 % prevalence in an autopsy series (1, 2). They occur around the third to fifth decade of life with male predominance. The diagnosis of pericardial angiosarcoma is difficult, since the presentation is generally non-specific and imaging modalities, such as echocardiography and computer tomography (CT) scan are nonspecific (3). The tumor is very aggressive and has a poor prognosis. Frequently patients are misdiagnosed with the ischemic heart disease or viral pericarditis and the diagnosis is made late. Tissue diagnosis remains most accurate, and immunohisto-chemical procedures can complement conventional tissue biopsy and helps in clinching the diagnosis (1). When the diagnosis is made early, surgical resection and chemotherapy is recommended as the treatment choice. The mean survival is approximately 6 months even after aggressive treatment (3, 4). Primary cardiac epithelioid angiosarcoma is extremely rare to our knowledge. Herein, we present a case of a primary pericardial epithelioid angiosarcoma.

## Case Presentation

We report a 16-year-old female who presented to our institute with chest pain and dyspnea on exertion for one month. On admission, transthoracic echocardiography revealed a localized pericardial effusion. However, no obvious mass was identified either in the pericardium or in the heart. Left ventricular ejection fraction was normal (60%). 

Pericardiocentesis and drainage were performed with draining 800 mL of pericardial effusion. The patient’s symptoms improved after drainage of pericardial ﬂuid. Her dyspnea and pericardial effusion recurred after one month. On physical examination, her pulse rate, respiration rate, and blood pressure were 128 beats/min, 22/min, and 135/75 mmHg, respectively. An echocardiogram demonstrated pericardial tamponade, LV ejection fraction was 55%, visceral and parietal pericardium were irregular, with thickening. Constrictive pericarditis was more likely to be the diagnosis according to the cardiac ultrasound, and for this reason, the patient was taken to the operating room for pericardiectomy.

**Fig. 1:**
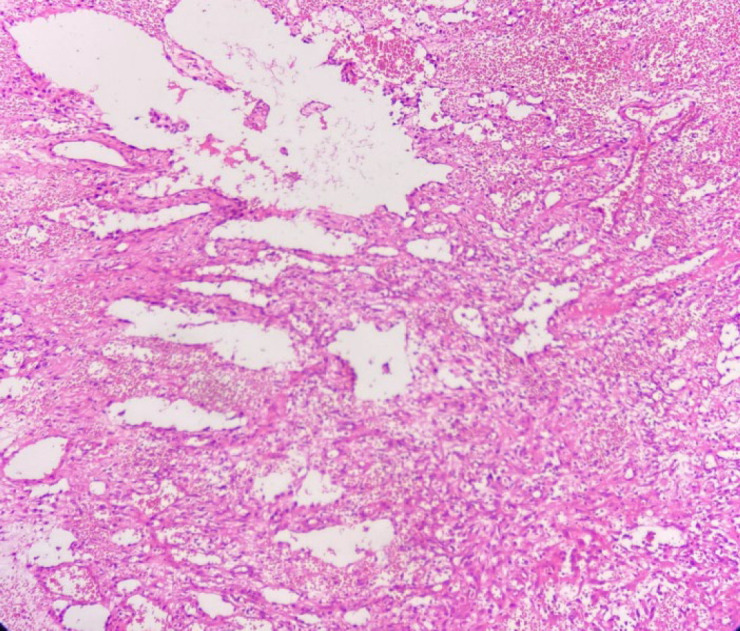
H&E stain (Objective 10x). Irregular blood vessels are surrounded by neoplastic cells in two different patterns: spindle cells fusing with each other and cells forming nests

**Fig. 2:**
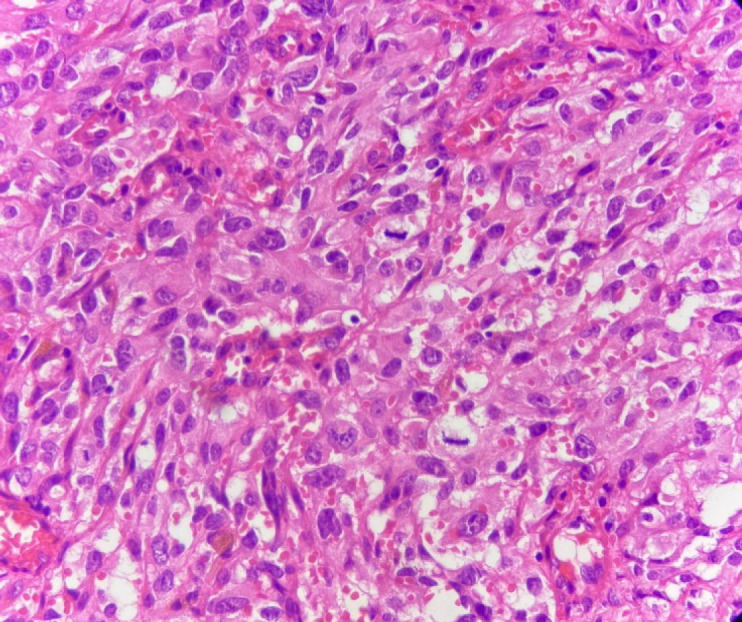
H&E stain (Objective 40x). The tumoral cells exhibit medium to large size vesicular nuclei, prominent nucleoli, eosinophilic cytoplasm and numerous mitotic figures

**Fig. 3:**
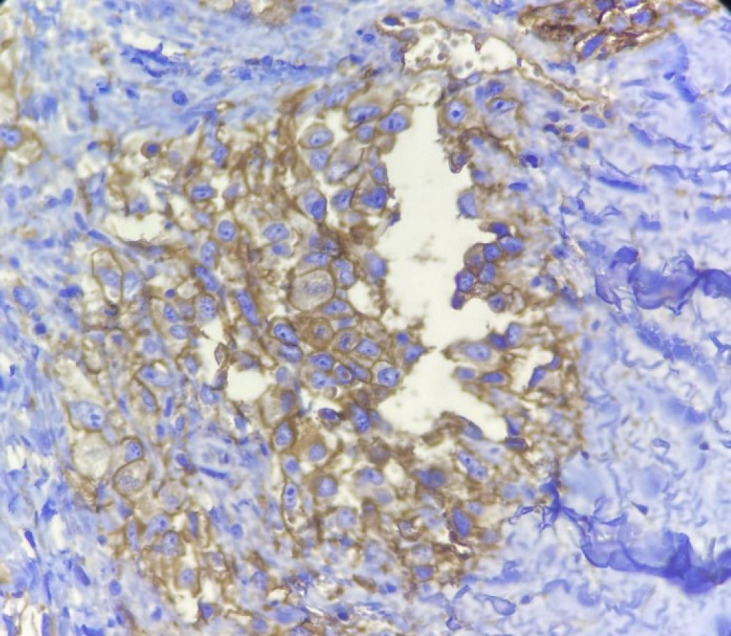
Immunohistochemical staining for CD31 marker. (Objective 40x). Tumoral cells show cytoplasmic and membranous positivity for CD31

Macroscopically, the specimen was a 5× 2.5× 2 cm soft brown tissue with no identifiable mass lesion. Histopathologic examination showed polygonal cells and some spindle-shaped cells arranged around the intercommunication blood vessels. The solid areas of epithelioid anaplastic cells with large pleomorphic, vesiculated nuclei, prominent nucleoli and abundant mitotic activity were present (Figures 1 and 2). The vascular channels were lined by atypical spindle to plump cells. Some foci of hemorrhage and necrosis were also observed. Through immunohistochemical analysis, neoplastic cells demonstrated intense positive reactivity for CD31 and vimentin. Ki67proliferative index was 20% (Figure 3). A positive cytoplasmic immunoreactivity in tumor cells for WT1 was detected. Reactive mesothelial cells revealed positive nuclear staining for later marker in the same sections. Hence, the overall findings were consistent with pericardial angiosarcoma, epithelioid variant. After the operation, the patient received three cycles of chemotherapy with adriamycin, ifosfamide, and mesna. Unfortunately, she finally died of multiple pleural metastases and progressive cardiac invasion less than three months following the operation.

## Discussion

Angiosarcoma of the pericardium is a rare malignancy, and generally of a rapid progressive course. The prognosis is generally very poor even if the diagnosis is made at an early stage. Diagnosis and treatment of this kind of angiosarcoma would be challenging for clinicians because it often remains clinically silent until the disease is advanced. Most cases presenting with cardiac tamponade can be easily mistaken with constrictive pericarditis (5). Echocardiography, although frequently performed, plays a limited role in the evaluation of primary pericardial angiosarcoma. Echocardiography frequently demonstrates pericardial effusion but may not show a mass because it depends on good acoustic window (6). Despite all imaging advances, tissue diagnosis still remains the gold standard. All pathology analyses should include immunohistochemistry, and a large panel of antibodies must be performed to avoid misdiagnosis. The differential diagnosis includes malignant mesothelioma, synovial sarcoma and metastatic carcinoma. The tumoral cells stain positively for CD34, CD31, factor VIII-related antigen, WT-1, vimentin and high Ki67 labeling (1, 7). Cardiac angiosarcomas usually have a poor prognosis; those tumors most frequently affect men aged 40 to 50 years and prognosis is worse in younger patients, as our patient we presented here. The mean survival rate of cardiac angiosarcomas is 9.3 ± 4.2 months without surgical treatment (8). The survival time is short compared to that of other cardiac sarcomas. Surgical resection with or without adjuvant radiation or chemotherapy is the main treatment modality. But the outcome may vary. The most common chemotherapeutic drugs are adriamycin, ifosfamide, cyclophosphamide, vincristine, and dacarbazine (5). In our case, the patient received three cycles of chemotherapy with adriamycin, ifosfamide, and mesna after operation. Unfortunately, she died due to the multiple pleural metastases less than three months following the operation which confirms a highly aggressive behavior, and rapid progression encountered in this type of tumor.

## Conclusion

We described a case of a patient with primary pericardial epithelioid angiosarcoma who presented with recurrent pericardial effusion and rapidly progression in a short period. Our case indicated that unexplained pericardial effusion in younger patients should prompt the clinician to seek for a malignant process etiology. Echocardiography, although frequently is performed, could be associated with limited utility in the evaluation of primary pericardial angiosarcoma. Tissue biopsy and immunohistochemical assessment is required to confirm the final diagnosis.
